# Evaluation and entomopathogenicity of gut bacteria associated with dauer juveniles of *Oscheius chongmingensis* (Nematoda: Rhabditidae)

**DOI:** 10.1002/mbo3.823

**Published:** 2019-03-27

**Authors:** Jun‐rui Fu, Qi‐zhi Liu

**Affiliations:** ^1^ Laboratory of Entomology and Nematology Department of Entomology College of Plant Protection China Agricultural University Beijing China

**Keywords:** *Bacillus cereus*, entomopathogenicity, high‐throughput sequencing, *Ochrobactrum tritici*, *Oscheius chongmingensis* Tumian

## Abstract

The nematodes of genus *Oscheius* are insect parasites with a potential role as biological control agents. The composition of gut microbiota and its potential assistant role in the complex pathogenic mechanism of nematodes have been poorly illustrated. In this study, the intestinal bacteria associated with dauer juveniles of the nematode *Oscheius chongmingensis* Tumian were classified by 16S rDNA high‐throughput sequencing. The raw reads were assigned to 845 operational taxonomic units (OTUs) after quality filtering. The results showed that the genus *Ochrobactrum*, with a proportion of 59.82%, was the most abundant genus, followed by 7.13% *Bacillus*, 4.7% *Albidiferax*, 4.26% *Acinetobacter*, and 3.09% *Rhodococcus*. The two dominant bacteria, *Ochrobactrum* and *Bacillus*, were further isolated by culturing on NBTA and LB medium respectively, and then identified as *Ochrobactrum tritici* and *Bacillus cereus* by morphological and 16S rDNA sequence analysis. Furthermore, the entomopathogenicity of these two bacterial species was studied. The results showed that *O. tritici* caused 93.33% mortality within 144 hr in the 4^th^‐instar larvae of *Galleria mellonella* treated with 2 × 10^9^ CFU/ml, whereas *B. cereus* showed 100% mortality at a concentration of 3.3 × 10^7^ CFU/ml within 48 hr. These findings, especially the presence of *O. tritici*, which had not been found in other nematode species in the genus *Oscheius*, indicate that the associated nematode *O. chongmingensis* may have particular utility as a biocontrol agent.

## Graphical abstract

The nematodes of genus *Oscheius* are insect parasites with a potential role as biological control agents. We identified and analyzed the gut bacterial community of the dauer juveniles of *O. chongmingensis* Tumian.

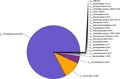

## 1 INTRODUCTION

Nematode species in the genus *Rhabditis* (*Oscheius*) have been characterized as entomopathogenic nematodes (EPNs) (Poinar,^1979^; Schulte, 1989; Smart & Nguyen, 1994). Recent studies have reported that some rhabditid nematodes could act as potential biocontrol agents to control various invertebrate pests, such as the burrower bug (Stock, Caicedo, & Calatayud, 2005), Formosan subterranean termite (Carta & Osbrink, 2005), areca nut spindle bug (Mohandas, Sheeba, Firoza, & Rajamma, 2007), rice yellow stem borer (Padmakumari, Prasad, Katti, & Sankar, 2007) and burying beetle (Sangeetha, Rajitha, Shyni, & Mohandas, 2016).

The dauer juvenile of entomopathogenic nematodes is the only infective juvenile stage (IJs) that invades insect hosts, and releases bacteria to produce toxins (Dillman et al., 2012). Furthermore, entomopathogenic nematodes are highly virulent due to the symbiotic bacteria found in IJs. In addition, entomopathogenic bacteria are promising sources of antimicrobial, insecticidal and nematicidal compounds, which might become potential biopesticides. Some nematodes of *Oscheius* sp. have been found to associate with insect pathogenic bacteria. However, the diversity of bacteria associated with nematodes of *Oschieus* sp. differs from the specific bacterial symbionts of the genera *Heterorhabditis* and *Steinernema*. Some reports have shown that the some species of intestinal bacteria associated with rhabditid nematodes could serve to promote nematode biological control effects. Nineteen bacterial genera have been found to be mutually associated with *Rhabditis* (*Oscheius*) nematodes: *Bacillus* (Mohandas et al., 2007), *Klebsiella*,* Acinetobacter*,* Comamonas*,* Brucellaceae*,* Achromobacter* (Deepa, Mohandas, & Siji, 2011), *Alcaligenes*,* Flavobacterium*,* Providencia* (Park, Kim, Ha, & Youn, 2011), *Stenotrophomonas*,* Enterobacter*,* Proteus*,* Pseudomonas*,* Enterococcus*,* Lysinibacillus* (Padmakumari et al., 2007; Sangeetha et al., 2016), *Microbacterium, Serratia, Rheinheimera* and *Staphylococcus* (Tambong, 2013). Among them, *Bacillus cereus* isolate 03BB102 (Mohandas et al., 2007), *Flavobacterium* sp., *Providencia vermicola* (Deepa et al., 2011), *Serratia marcescens* (Tambong, 2013) and *Serratia nematodiphila* (Zhang, Yang, Xu, & Sun, 2009) exhibit virulence against insects and help improve nematode reproduction.

Notably, *S. nematodiphila* was associated with the nematode species *O. chongmingensis*, which was collected from the soil of Chongming Island in the southeastern area of Shanghai, China (Zhang et al., 2008). In the present work, the same nematode species, the *Oscheius chongmingensis* Tumian strain, isolated from an alfalfa field in the city of Hailar, Inner Mongolia, China (Liu, Mráček, Zhang, & Půža, 2012), the presence of the same or different associated bacterial genera in its intestine was assessed. In previous studies, dauer juveniles of *O. chongmingensis* Tumian showed pathogenicity to *Galleria mellonella* and *Tenebrio molitor* (Cao, Liu, Xie, Cao, & Li, 2007), and this nematode strain also led to 90% mortality of the longhorn beetle, *Batocera lineolata* (Coleoptera: Cerambycidae), which attacks walnut trees in walnut fields (Liu & Wei, 2015). Hence, *O. chongmingensis* Tumian has been identified as a potential biocontrol agent.

Therefore, in the present study, the intestinal bacteria associated with the dauer juveniles of *O. chongmingensis* Tumian were investigated and the effects of two associated bacteria on pathogenicity were analyzed. The results will benefit further study of the infection mechanism of this nematode against insect pests.

## 2 MATERIALS AND METHODS

### 2.1 Nematode culture and DNA extraction

*Galleria mellonella* larvae were reared in the Entomology and Nematology Laboratory, Department of Entomology, China Agricultural University (CAU), using artificial diets (Pu & Liu, 2009). The nematode *O. chongmingensis* Tumian was originally isolated from a soil sample collected from an alfalfa field in the city of Hailar, Inner Mongolia, using the Galleria bait method. The nematode has been subsequently maintained through reinfections of fourth‐instar larvae of *G. mellonella* (Lepidoptera: Galleridae). To produce fresh nematodes for the study, *G. mellonella* larvae were infected with *O. chongmingensis* Tumian at room temperature and then transferred to White traps to facilitate the harvest of dauer juveniles. The nematodes were suspended in distilled water and counted. Only newly emerged IJs (<48 hr) were utilized for the inoculation test in each experiment.

The freeze‐thaw lysis protocol was adopted to extract genomic DNA from 100 dauer juveniles of *O. chongmingensis* Tumian. The nematodes were surface‐sterilized by immersion in 1% (w/v) NaClO (sodium hypochlorite solution) for 30 min and then in 75% alcohol for 1 min, followed by washing three times with sterile water. To avoid contamination by foreign bacteria, 200 μl of the treated nematode suspension was dropped onto Luria‐Bertani (LB) plates and incubated for 24 hr. Only nematode samples with no bacterial growth on LB were used for further analysis. Total DNA from dauer juvenile nematodes was extracted using a Power Soil^®^DNA Isolation Kit (MoBio) according to the manufacturer's instructions. All operations were carried out under a sterile laminar flow hood. Purified DNA extracts were stored at −20°C until PCR amplification.

### 2.2 PCR amplification and high‐throughput sequencing

DNA fragments of the bacterial 16S rDNA gene, targeting the hypervariable region V3–V4, were amplified using the primer pairs 336F (5′‐GTACTCCTACGGGAGGCAGCA‐3′) and 806R (5′‐GTGGACTACHVGGGTWTCTAAT‐3′). PCR amplification was carried out in triplicate 50 μl reactions containing 30 ng of genomic DNA, 0.3 μl of *Pyrobest* DNA Polymerase (2.5 U/μl, TaKaRa), 2 μl of each barcoded fusion primer (10 μM), and 4 μl of dNTPs (2.5 mM), in the appropriate 10 × *Pyrobest* Buffer and ddH_2_O. Negative control samples were treated similarly with the exclusion of template DNA. The following thermal program was used for amplification: an initial denaturation at 95°C for 5 min, followed by 25 cycles of denaturation at 95°C for 30 s, annealing at 56°C for 30 s, and extension at 72°C for 40 s, with a final extension step at 72°C for 10 min. The triplicate PCR products were pooled and purified from 2.0% agarose gels. The amplicon products were quantified by the Qubit fluorescence quantitative system and then combined in an equimolar ratio in a single tube. Paired‐end sequencing was performed on the Illumina platform (MiSeq) (Allwegene Technology Co., Ltd., Beijing, China).

### 2.3 Analysis of data processing and bacterial diversity

The data were processed by removing low‐quality reads using Trimmomatic software and Readfq (version 6.0) and then splicing the paired reads into a sequence based on the PE data by FLASH (version 1.2.10) and Pear software. Sequences with lengths shorter than 200 bp or with maxhomop >10 were removed using the Mothur pipeline (Schloss, Westcott, Ryabin, & Hall, 2009). Sequences with ambiguous bases, primer mismatches, errors in barcodes, and chimerism were filtered using Usearch (version 8.0.1623) (Edgar, Haas, Clemente, & Quince, 2011). The remaining high‐quality sequences were aligned to a reference alignment derived from the 16S rDNA gene database (DeSantis, Hugenholtz, Larsen, & Rojas, 2006). Then, the spliced and filtered clean tags were clustered into OTUs (Operational Taxonomic Units) using a 3% distance cutoff by using the QIIME pipeline (v.1.8.0) (Caporaso, Kuczynski, Stombaugh, & Bittinger, 2010; Schloss & Westcott, 2011).

To improve the accuracy of the annotation, sequences were also searched against the NCBI nt database using BLASTn (Altschul, Gish, Miller, & Myers, 1990). Taxonomic information was assigned by the NCBI database according to the highest scoring sequence. The diversity index and species richness estimate were calculated using the Mothur pipeline. The α‐diversity was represented by rarefaction curves plotting the cumulative number of OTUs at a 3% distance level. Diversity was measured by counting the number of observed OTUs using the Shannon index, Chao1 index, phylogenetic diversity and observed number of species as described previously (Magurran, 2004; Chao, 1984). The estimators were calculated by subsampling the smallest number of sequences from each sample. Statistical analysis was performed in R (version 3.11) (R Development Core Team, 2014). The dominant taxonomic unit, species richness and relative abundances in each sample were determined from rank‐abundance curves. Beta diversity and species composition analysis were constructed by using the QIIME pipeline and R software.

### 2.4 Isolation of dominant bacteria from nematodes

Associated bacteria were obtained from the infective stages of *O. chongmingensis* Tumian by two methods. Dauer juveniles of nematodes were surface‐sterilized by immersion in 1% (w/v) NaClO (sodium hypochlorite solution) for 30 min and then in 75% alcohol for 1 min; after washing three times in sterile water, they were streaked onto nutrient agar NBTA (peptone, 10 g; beef extract, 5 g; NaCl, 5 g; agar, 18 g; water, 1 L, 0.0025% bromothymol blue, 0.004% triphenyltetrazolium chloride; pH 7.0–7.2) and LB (tryptone, 10 g; NaCl, 10 g; yeast extract, 5 g; water, 1 L; pH 7.0–7.2). In addition, associated bacteria were isolated from the hemolymph of dead *G. mellonella* larvae infected with the nematode species. The dead larvae were surface‐sterilized by dipping into 75% ethanol for 1 min and placed in a sterile petri dish to dry. Sterile scissors were used to dissect the 3^rd^ segment from the head of each larva. A sterile loop was used to touch the hemolymph and streak it on the nutrient agar media. The plates were sealed and incubated in the dark at 28°C for 48 hr. Developing bacterial colonies that differed in morphology or in color were transferred to NBTA or LB plates (Akhurst, 1980). Before and after each test, cultures were streaked onto NBTA and LB to confirm that there had been no changes from one form to the other.

These colonies were added to LB nutrient broth and shaken for 48 hr (150 rpm) at 28°C in the dark. Subsequently, the bacterial suspension was centrifuged at 4,000 *g* for 8 min and the supernatant decanted. Sterile water was added to the pellets and mixed thoroughly to obtain a concentrated suspension of the bacterial symbionts. The total number of bacteria in the suspension was measured with a spectrophotometer with a wavelength at 600 nm. The cell number in the suspension was estimated by counting colonies on culture plates of different concentration gradients and measuring the OD_600_ value. Some of the colonies were sampled for molecular analysis.

### 2.5 Morphology and identification of bacterial isolates

Cultural properties such as colony size, shape, and color were observed after 48 hr incubation at 28°C on nutrient agars (NBTA and LB), and then, the bacteria were gram stained.

Pure isolates of two strains, NMA‐1 and NMA‐2, were used for identification following 16S rDNA gene sequencing. For DNA extraction, bacterial isolates were grown individually in 5 ml of LB broth at 28°C for 24 hr. DNA was extracted following the procedure described previously (Krsek & Wellington, 1999) with some modifications. The sample was suspended in 100 μl 1 × TE (pH 8.0) and 100 μl of lysozyme solution (50 mg·mL^−1^) was added. The tube was placed in a 37^o^C water bath for 20 min. Subsequently, 100 μl of 10% SDS solution (10% w/v SDS) and 5 μl of proteinase K (20 mg·mL^−1^) were added, and the contents of the tube were gently mixed and placed in a 37^o^C water bath for 30 min. Then, 100 μl of NaClO solution (5 M) was added, and the mixture was gently whirled for 1 min; the supernatant was treated with phenol:chloroform:isoamyl alcohol (25:24:1) and chloroform:isoamyl alcohol (24:1) successively to remove proteins and other impurities. Thereafter, the DNA was precipitated with 90 μl of isopropanol for 10 min at room temperature and then pelleted by centrifugation at 12,000 *g* and 4°C for 10 min. The precipitate was washed with 75% ethanol and dried in a laminar flow cabinet for 1 hr prior to resuspension with 50 μl of sterile water. DNA extracts were stored at −20°C until further analysis.

The gene sequences were amplified using three different primer sets: two targeting the bacterial 16S rDNA gene and a second pair targeting a different gene, recA (Table 1). The primer sets for bacteria were 27f/1492r and S1/A1 (Yang, 2008) (Table 1), while the primer set for the *Brucella* spp. recA gene was recA‐f/recA‐r (Scholz, Pfeffer, Witte, & Neubauer, 2008). PCR amplification was performed in a thermal cycler (Bio‐Gener) using approximately 1.0 μl of bacterial DNA, 2.5 μl of 10 × PCR buffer, 2.0 μl of 2.5 mM dNTPs, 1.0 μl of each primer (10 μM), 0.3 μl of Taq DNA polymerase, and 17.2 μl of ddH_2_O. Thermal cycling conditions were as follows: 3 min at 95°C, followed by 35 cycles of 30 s at 95°C, 30 s at 55°C for 27f/1492r and recA‐f/recA‐r or 1 min at 63°C for S1/A1, 1.5 min at 72°C, and a final step at 72°C for 10 min. The PCR products were sequenced by BGI (Beijing, China).

**Table 1:** Primer pairs used for identification in this study

Gene	Primer	Sequence (5′ → 3′)	Tm (°C)
16S rDNA	27F	AGAGTTTGATCCTGGCTCAG	55
	1492R	GGTTACCTTGTTACGACTT	
	S1	GAAGAGTTTGATCATGGCTC	63
	A1	AAGGAGGTGATCCAGCCGCA	
recA gene	recA‐f	ATGTCTCAAAATTCATTGCGAC	54
	recA‐r	AGCATCTTCTTCCGGTCCGC	

### 2.6 Sequence and phylogenetic relationship analysis of bacteria

For phylogenetic analysis, all nucleotide sequences obtained were compared with NCBI database sequences using BLASTn. The selected sequences were aligned using ClustalW, and neighbor‐joining trees of the homologous sequences were constructed using the maximum likelihood method with 1,000 bootstrap replications in the MEGA 7.0 program (Kumar, Steche, & Tamura, 2016).

### 2.7 Physiological and biochemical characteristics of bacterial isolates

Physiological and biochemical tests were performed at 28°C according to *Bergey's Manual of Determinative Bacteriology* (Buchanan & Gibbons, 1986) and *Common Bacterial System Identification Manual* (Dong, Cai, Lu, Xie, & Liu, 2001).

### 2.8 Evaluation of potential pathogenicity of bacterial isolates to *G. mellonella* larvae

The two species of bacteria were cultured separately for 48 hr, concentrated, and rinsed three times with sterile water as previously described (Delalibera, Handelsman, & Raffa, 2005). Serial dilutions were performed in sterile water to obtain dilutions from 1 × 10 to 1 × 10^6^, which corresponded to suspensions of 2.0 × 10^5^–2.0 × 10^10^ CFU/ml for the strain NMA‐1 and 3.3 × 10^2^–3.3 × 10^7^ CFU/ml for the strain NMA‐2. In addition, 100 μl of serial dilutions was streaked onto LB agar plates to count the number of colonies, and the OD_600_ values of gradient dilutions were measured by an ultraviolet‐visible light detector (LabTech) to make a standard curve. *G. mellonella* larvae were used as the test insect for assessing the pathogenicity of the two dominant bacteria associated with *O. chongmingensis* Tumian. A cell suspension (50 μl) was injected into 4^th^‐instar *G. mellonella* larvae using a 50 μl Anting microsyringe (Shanghai, China). Sterilized water was used as a control. Thirty larvae were used per concentration, and three replications were performed. Insect mortality was observed every 24 hr after injection.

## 3 RESULTS

### 3.1 Basic data processing and statistics

16S rRNA gene sequencing of the dauer juvenile nematode samples was performed by paired‐end sequencing. After the quality check, unqualified sequences shorter than 200 bp were removed. According to the quality criteria, approximately 97.47% of the raw sequences were used for subsequent analysis (Table 2). Next, filtering processes were performed to remove edundant, chimeras and undesirable sequences, generating 51,651 clean reads (91.69%) for dauer juveniles. After rarefying the reads to the smallest number, 51,651 bacterial sequences from nematodes were retained.

**Table 2:** Sequence statistics

Sample	Raw tags	High‐quality tags		Clean tags	
		Number	%^a^	Number	%^a^
Dauer Juvenile	56,334	54,907	97.47	51,651	91.69

a Percent of raw reads.

### 3.2 Diversity and richness of bacterial species

The Chao1 index was used to analyze OTU richness and species richness at the 0.03 dissimilarity level. The shannon index, phylogenetic diversity (PD, whole tree) and observed number of species were used to assess the diversity of the bacterial communities from nematodes. Similar results were obtained (Table 3). The Shannon index of the nematode sample was 3.60, and the value of Good's coverage was 0.99. In addition, the value of the PD whole tree was 65.2 (Table 3). These results suggested that dauer juveniles of *O. chongmingensis* Tumian had high bacterial diversity.

**Table 3:** Species richness estimator and diversity index of bacterial community

Sample	OTUs	Chao1	Goods_coverage	Observed_species	PD_whole_tree	Shannon
Dauer juvenile	845	924.61	0.99	766	65.20	3.60

### 3.3 Analysis of bacterial community structure and predominant species

**Figure 1:**
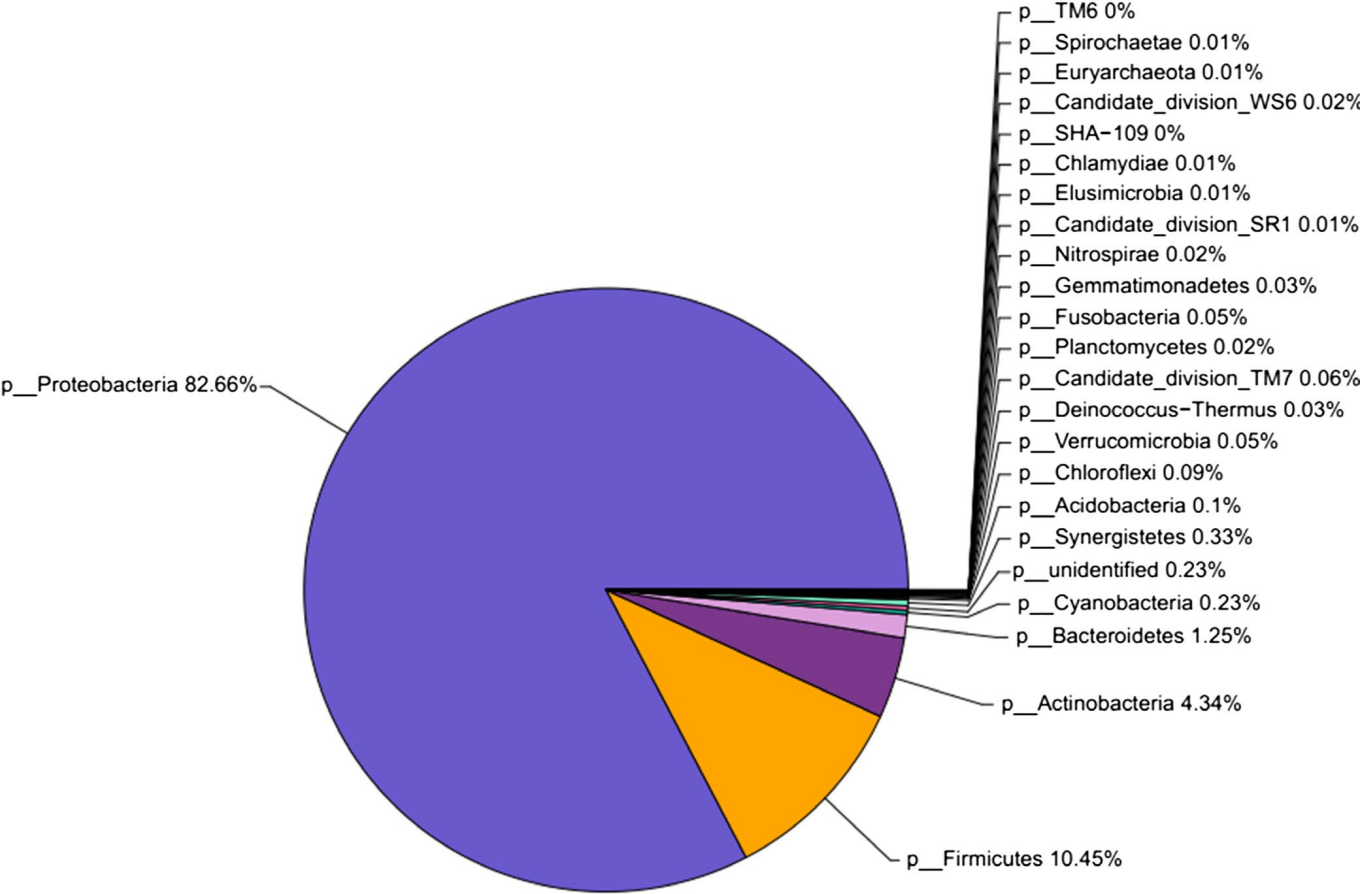
The composition and relative abundances of bacterial phyla associated with the dauer juveniles of *Oscheius chongmingensis* Tumian. Only OTUs with at least 20 sequences are represented[Colour figure can be viewed at wileyonlinelibrary.com]

The 845 OTUs from the nematode sample were assigned to corresponding taxonomic groups based on the combined search results from the Greengenes and NCBI databases. The relative abundances of different phyla in the sample are shown in Figure 1. The results showed that Proteobacteria was the most heavily sequenced phylum associated with the dauer juvenile nematodes (82.66%). Firmicutes (10.45%), Actinobacteria (4.34%) and Bacteroidetes (1.25%) were also present in the juvenile communities.

**Figure 2:**
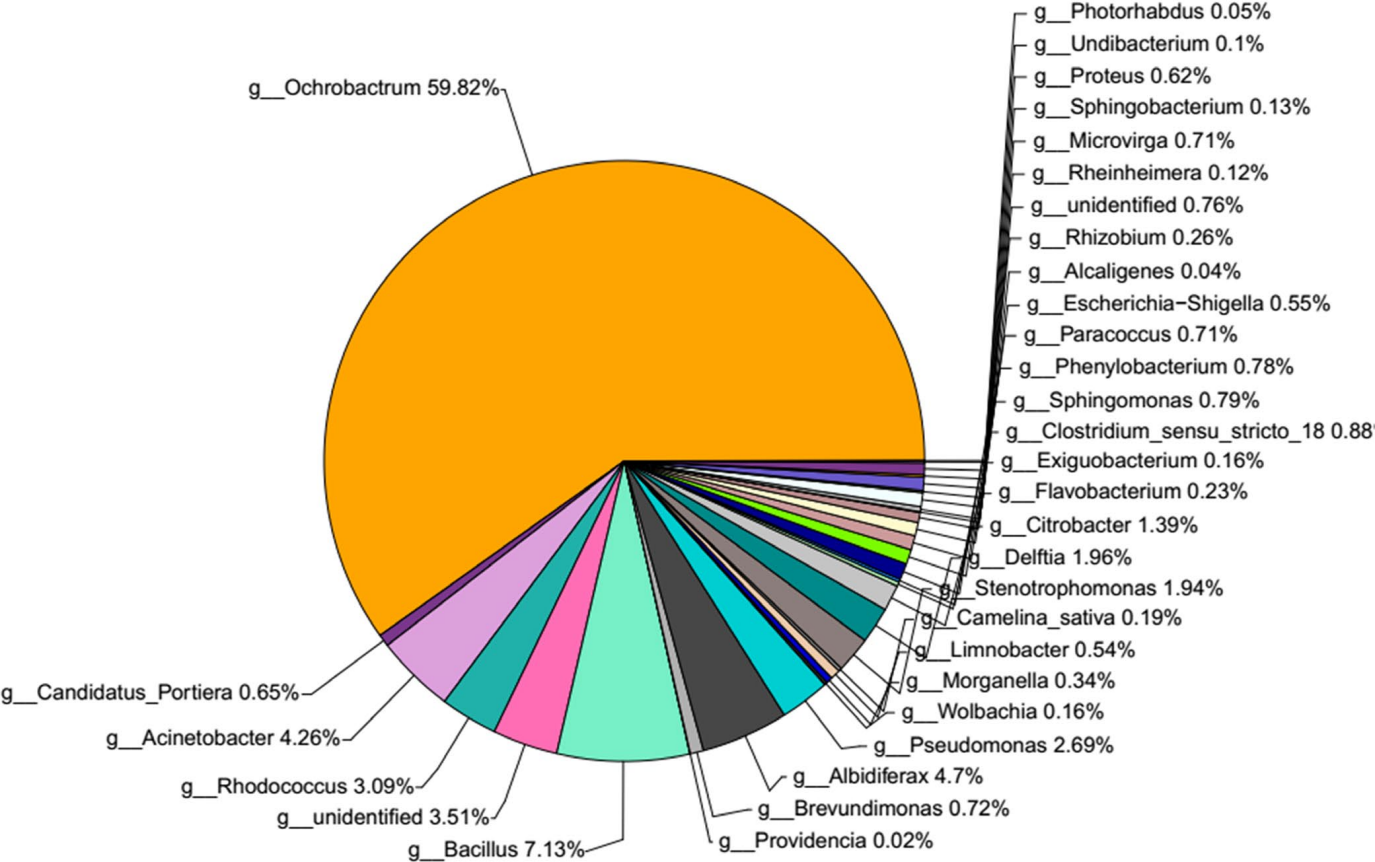
The composition and relative abundances of bacterial genera associated with the dauer juveniles of *Oscheius chongmingensis* Tumian

Under the 97% similarity level, the trimmed clean tags were clustered into OTUs for species classification by QIIME (v1.8.0) software. To describe the majority of bacterial genera, OTUs of the nematode samples were assigned to corresponding taxonomic groups based on the combined search results from the NCBI databases. *Ochrobactrum* was the most abundant bacterial genus detected in dauer juveniles of *O. chongmingensis* Tumian, with an abundance of 59.82%, followed by *Bacillus* (7.13%), *Albidiferax* (4.7%), *Acinetobacter* (4.26%), *Rhodococcus* (3.09%), *Pseudomonas* (2.69%), *Delftia* (1.96%), *Stenotrophomonas* (1.94%), and *Citrobacter* (1.39%). All other genera associated with dauer juveniles are shown in Figure 2.

### 3.4 Morphological characteristics of bacteria

**Figure 3:**
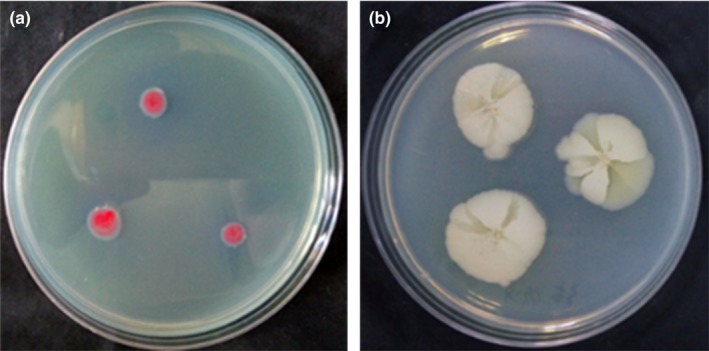
Colony morphology of the two main bacteria species isolated from *Oscheius chongmingensis* Tumian on nutrient agar culture. (a) NMA‐1 strain on NBTA. (b) NMA‐2 strain on LB

Two bacterial species were isolated from surface‐sterilized nematodes and from the hemolymph of *G. mellonella* larvae infected by the nematodes. After growth on NBTA plates for 48 hr, the NMA‐1 strain was obtained and distinguished as gram‐negative (Figure 3a). During incubation on NBTA, the pink colonies of NMA‐1 were opaque, mucoid, smooth, protuberant and rapidly confluent. The gram‐positive NMA‐2 strain was obtained on LB nutrient agar after 24 hr (Figure 3b). The colonies of the NMA‐2 strain were large (approximately 15 mm in diameter), and white waxy with a rough surface and a flat, irregular shape.

### 3.5 16S rDNA sequence and phylogeny of two dominant bacteria

**Figure 4:**
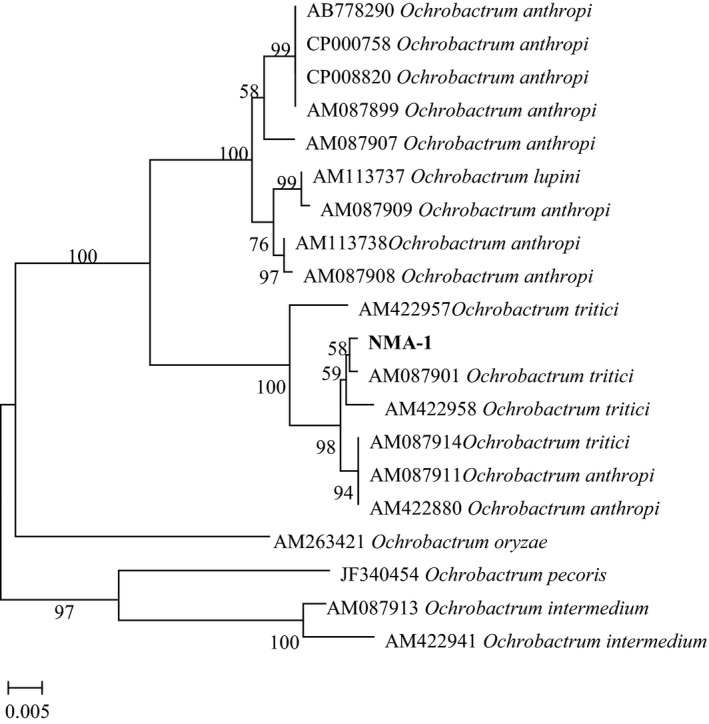
Phylogenetic relationships of the strain NMA‐1 and other closely related *Ochrobactrum* species in a neighbor‐joining tree based on analysis of the recA sequence. Bootstrap values are 1,000 replications and above 50% are shown at the branch points by MEGA 7.0

**Figure 5:**
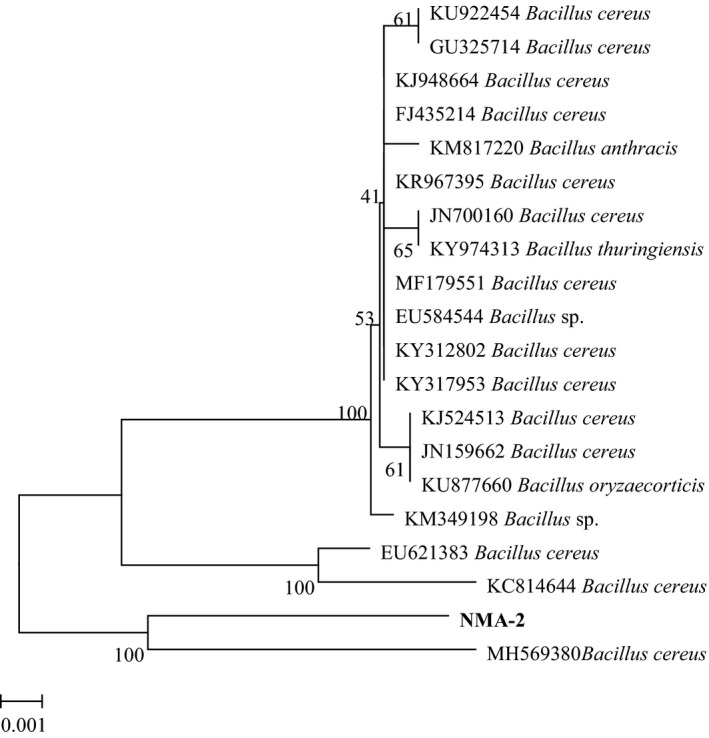
Phylogenetic tree of strain NMA‐2 and other related *Bacillus* species in a neighbor‐joining tree based on analysis of the S1/A1 sequence data. Bootstrap values are 1,000 replications and above 50% are shown at the branch points by MEGA 7.0

The associated bacteria were confirmed by molecular analysis based on the comparison of their 16S rDNA. Strain NMA‐1 was 99% homologous to *O. tritici*, and strain NMA‐2 was *B. cereus* with 100% identity. The analysis of morphological characteristics and 16S rDNA sequencing showed conclusively that strain NMA‐1 belonged to *O. tritici* and strain NMA‐2 was close to *B. cereus*. The phylogenetic relationship analysis (Figure 4) demonstrated that the genetic distance between the amplified fragment and *O. tritici* (GenBank accession no. AM087901) was the smallest, and they were in the same clade (with a confidence level of 88%). Alternatively, NJ trees (Figure 5) based on the S1/A1 sequence showed that all 17 *B. cereus* isolates belonged to a single cluster, including strain NMA‐2 (with 86% bootstrap support).

### 3.6 Physiological characteristics of two dominant bacteria

Phenotypic characteristics are shown in Table 4. Both strains assimilated D‐glucose, sucrose, D‐fructose, and D‐trehalose but not mannitol or lactose, and they produced urease, lecithinase and amylase. Furthermore, both strains hydrolyzed starch. Strain NMA‐1 produced indole, but did not hydrolyze gelatin, while strain NMA‐2 did both.

**Table 4:** Physiological and biochemical characteristics of strains NMA‐1 and NMA‐2

Characteristics	NMA‐1	NMA‐2
Glucose	+	+
Sucrose	+	+
Fructose	+	+
Trehalose	+	+
Mannitol	−	−
Lactose	−	−
Starch	+	+
Voges–Proskauer	−	−
Methyl red	+	+
Mycoderm	+	+
Gelatinase	−	+
Urease	+	+
Catalase	+	−
Arginine dihydrolase	−	−
Lecithinase	+	+
Aesculin	−	+
Tween 80	−	−
Malonate	−	−
Phenylalanine deaminase	−	−
Citrate	+	+
H_2_S	−	−
Indole	+	+
Litmus milk	+	+
Protease	−	+

+, Positive;−, Negative.

### 3.7 Pathogenicity of associated bacteria to *G. mellonella* larvae

**Figure 6:**
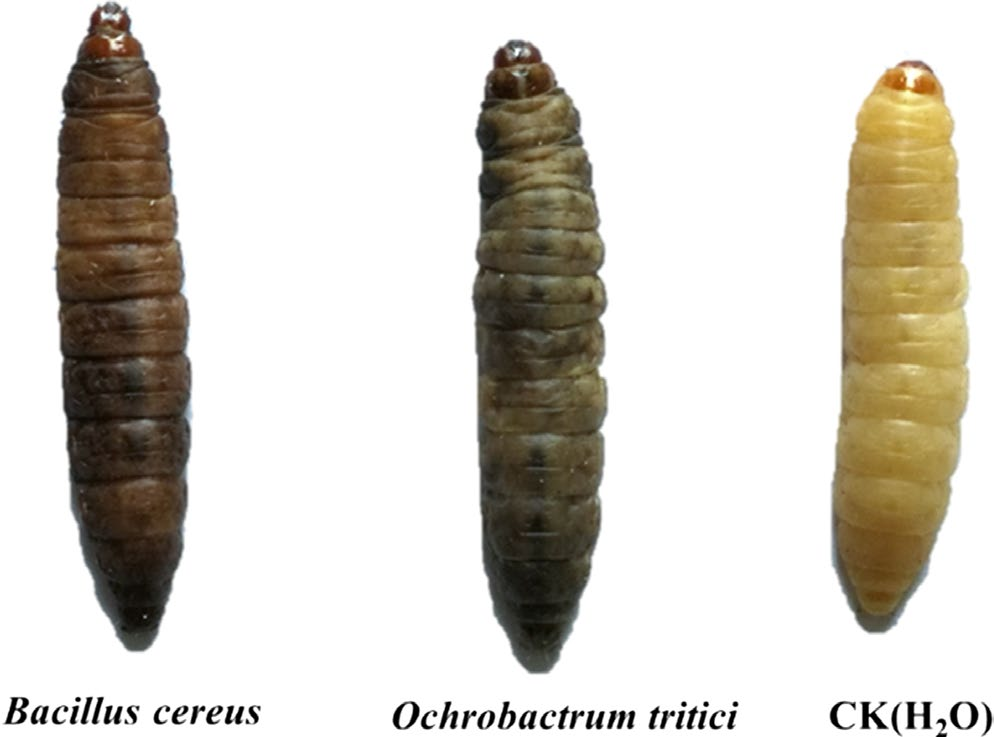
Death symptoms of *Galleria mellonella* larvae caused by injection of cell suspensions of *Ochrobactrum tritici* NMA‐1 and *Bacillus cereus* NMA‐2, sterile water as the control

To assess the ability of the nematode symbiotic bacteria to grow and survive within the host larval hemocoel and to test their pathogenicity, 50 μl of different bacterial suspensions was injected into *G. mellonella* larvae. Over time, the body color of the *G. mellonella* larvae became dark brown as the infected larvae died, while no changes were observed in the control (H_2_O) (Figure 6).

**Figure 7:**
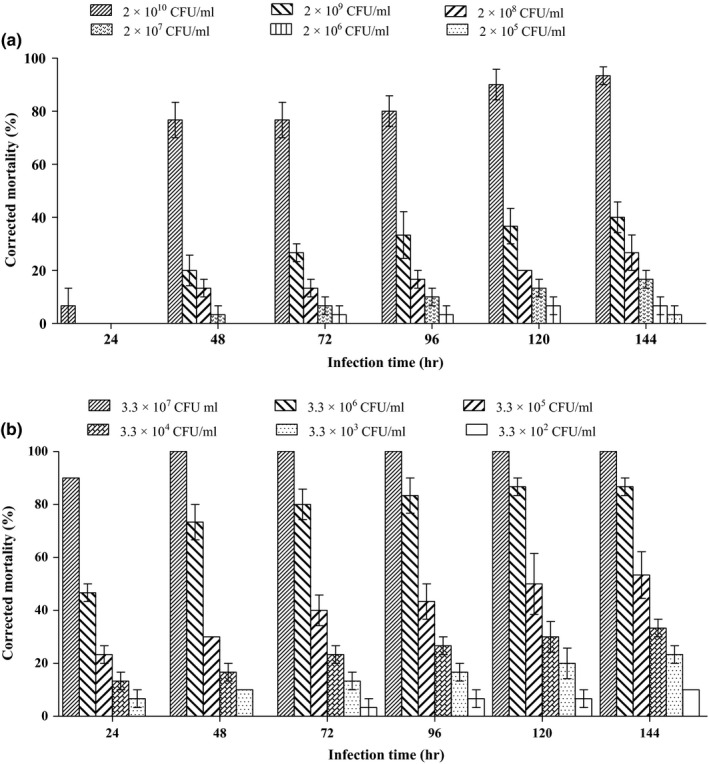
Corrected mortality of *Galleria mellonella* larvae injected with cell suspensions of (a) *Ochrobactrum tritici* NMA‐1 and (b) *Bacillus cereus* NMA‐2. Each data represents the mean of three replicates with 10 larvae per replicate and error bars standard errors of the means (*p* < 0.05; One‐way ANOVA, post hoc LSD)

The pathogenicity of serial dilutions of *O. tritici* and *B. cereus* at 48 hr after injection into the hemocoel of *G. mellonella* larvae is shown in Figure 7; a significant difference in insect mortality (*p* < 0.05) was observed between bacterial species. After 24 hr treatment, low insect mortalities of 0%–6.67% were observed for *O. tritici*, while 90% mortality caused by *B. cereus* was detected. Up to 120 hr after injection, larvae injected with the highest concentration (2.0 × 10^10^ CFU/ml) showed 90% mortality (Figure 7a). However, the highest mortality of larvae was 100% at 48 hr after injection of *B. cereus* NMA‐2 (3.3 × 10^7^ CFU/ml) (Figure 7b). Since *B. cereus* NMA‐2 was more pathogenic than *O. tritici* NMA‐1 after culture for 48 hr, it was possible that these bacteria had different characteristics and different effects on nematode reproduction and pathogenicity.

## 4 DISCUSSION

In the present work, we identified and analyzed the gut bacterial community of dauer juveniles of *O. chongmingensis* Tumian. We found gut microbiota with high diversity and two dominant bacterial OTUs. Comparison of the microbial composition revealed that the most abundant OTUs of the sequences belonged to the phyla (*Proteobacteria* (82.66%), *Firmicutes* (10.45%) and *Actinobacteria* (4.34%). Among them, the phylum *Proteobacteria* contains a variety of species, that are adapted to diverse environments (Delalibera et al., 2005). At least 33 bacterial genera were found to be associated with the dauer juveniles of *O. chongmingensis* Tumian, which was initially isolated from an alfalfa field in the city of Hailar, Inner Mongolia, China. The genus *Serratia,* as a biological control agent, has been found to associate with the nematode species *O. chongmingensis*, as well as some other nematodes, such as *O. carolinensis*,* O. myriophil*a and *O. rugaoensis* (Zhang et al., 2009; Liu et al., 2012; Ye, Torres‐Barragan, & Cardoza, 2010; Torres‐Barragan, Suazo, Buhler, & Cardoza, 2011; Liu, Zhang, & Zeng, 2016). However, no *Serratia* sp. was discovered in the *O. chongmingensis* Tumian strain in this report. Thus, differences in the dominant bacteria associated with rhabditid nematodes may be due to differences in the species of nematodes, isolation methods and bacterial species in the rhizosphere soil of different plants from different regions. Hence, different nematode habitats may affect their associated bacterial communities and, consequently, nematode lifestyle plasticity and pathogenicity.

The two highly abundant genera, *Ochrobactrum* and *Bacillus*, with high frequencies of separation, were further isolated by culture methods and identified as *O. tritici* and *B. cereus*, respectively. Both caused high mortality of *G. mellonella* larvae. In the genus *Ochrobactrum*, most members occur in the environment (soil, water, plant, human) (Jelveh & Cunha, 1999). Some species of *Ochrobactrum* can biodegrade polycyclic aromatic hydrocarbons (Arulazhagan & Vasudevan, 2011). Some endophytic bacteria belonging to *Ochrobactrum* sp. have been isolated from plants, and these strains exhibit maximum antagonistic activity and possess higher chitinase and glucanase activity (Zhao, Xu, Chang, & Li, 2016; Zang, Zhao, Liu, & Liang, 2014; Sowndhararajan, Marimuthu, & Manian, 2013). Metabolites produced by bacterial species of *Ochrobactrum* inhibit the growth of a wide range of bacteria and fungi (Han, Peng, & Yi, 2011; Lebuhn, Achouak, Schloter, & Berge, 2000). Only one report on one species in the genus, *O. anthropi*, has previously associated this genus with a nematode, *Steinernema scapterisci* (Aguillera, Hodge, Stall, & Smart, 1993). Nematodes in monoxenic culture with *O. anthropi* could result in 81% mortality to the southern mole cricket, *Scapteriscus borellii*. Until now, there had been no report on *Ochrobactrum* sp. in association with *Rhabditis* (*Oscheius*) sp. However, the strain *O. tritici* NMA‐1 was discovered and isolated from dauer juveniles of *O. chongmingensis* Tumian for the first time and showed high efficacy for controing *G. mellonella* larvae in this study.

The second most abundant genus investigated in this study was *Bacillus*. Previous studies had demonstrated that the bacterial species *B. cereus* was closely associated with different rhabditid nematodes (Deepa et al., 2011). The insecticidal proteins produced by *B. cereus* were significantly pathogenic to insects from the orders Lepidoptera and Diptera (Kaaya & Darji, 1989; Warren, Koziel, Mullins, & Nye, 1996). Moreover, some studies have revealed that bioactive metabolites produced by the strain of *B. cereus* associated with rhabditid nematodes cause insect mortality (Anju, Archana, Mohandas, & Nambisan, 2015a; Kumar, Nambisan, Sundaresan, & Mohandas, 2014; Nishanth, Nath, Pratap, & Nambisan, 2014). Similarly, the strain *B. cereus* NMA‐2 isolated from dauer juveniles of *O. chongmingensis* Tumian in the present study may have high potential insecticidal bioactivity.

The other most abundant genus detected in this study was *Acinetobacter*. One species of *Acinetobacter*,* A. baumannii*, is an opportunistic pathogen of great concern in nosocomial pneumonias and especially as an invader of burn wounds (Livermore & Woodford, 2006). *Acinetobacter calcoaceticus*, associated with the nematode *Steinernema* sp., produces compounds bioactive against *Candida albicans* (Reghunath, Siji, Mohandas, & Nambisan, 2017). In addition, the genus *Rhodococcus* has been found in the intestine and thrives in a broad range of environments, including soil, water, and eukaryotic cells. Two of the most important species were the plant pathogen *R. fascians* which causes leafy gall disease of gymnosperm plants and angiosperm (Goethals, Vereecke, Jaziri, & Montagu, 2001), and the human and animal pathogen *R. equi,* which infects foal pneumonia, cattle and immunocompromised humans (Muscatello, Leadon, Klayt, & Ocampo‐Sosa, 2007). Nevertheless, it was rare to isolate bacteria in the genera of *Acinetobacter* and *Rhodococcus* using nutrient agar in this study, although isolation was performed more than 10 times. These results suggest that *O. chongmingensis* Tumian could be a potential biocontrol agent, because it carries different pathogenic bacteria. Further studies are needed to understand the interactions between nematodes and associated bacteria in the natural environment.

Similar to *O. chongmingensis* Tumian, the nematode *Rhabditis blumi* also has more than one species of associated bacteria. The bacterium *Providencia vermicola* promotes was one of the bacteria that contributed to promoting nematode culture, in addition to *Flavobacterium* sp. Another species, *Alcaligenes faecalis,* however, is unfavorable for nematode reproduction and pathogenicity (Park et al., 2011). In addition, a slug‐parasitic nematode, *Phasmarhabditis hermaphrodita*, has been associated with multiple bacteria as well. Finally, the core bacterium *Moraxella osloensis* is the optimum species that can improve nematode production and pathogenicity (Wilson, Glen, George, & Pearce, 1995; Wilson, Glen, Pearce, & Rodgers, 1995).

Further studies are needed to analyze the function of *O. tritici*,* B. cereus* or other intestinal bacteria associated with *O. chongmingensis* Tumian to explore the mechanism of nematode infection pathogenicity to invertebrate pests.

## CONFLICT OF INTERESTS

The authors declare no conflicts of interest.

## AUTHORS CONTRIBUTION

Qi‐zhi Liu conceived and designed the experiments, confirmed the analyzed data and revised manuscript; Jun‐rui Fu performed the experiments, analyzed the data, drafted the manuscript and amended the revised manuscript according to the language company’s comments

## ETHICS STATEMENT

None required.

## Data Availability

All data are provided in full in the results section of this paper apart from the DNA sequences of *Ochrobactrum tritici* NMA‐1 and *Bacillus cereus* NMA‐2 genes which are available at https://submit.ncbi.nlm.nih.gov/subs/genbank/ under accession number MK434214 and MK434215, respectively.
